# Relationship between Angiotensin Converting Enzyme, Apelin, and New-Onset Atrial Fibrillation after Off-Pump Coronary Artery Bypass Grafting

**DOI:** 10.1155/2017/7951793

**Published:** 2017-02-12

**Authors:** Shu Xu, Jian Zhang, Yin-li Xu, Hai-bo Wu, Xiao-dong Xue, Hui-shan Wang

**Affiliations:** ^1^Department of Cardiovascular Surgery, Xijing Hospital, Fourth Military Medical University, Xi'an, Shaanxi 710032, China; ^2^Department of Cardiovascular Surgery, General Hospital of Shenyang Military Region, No. 83, Wenhua Road, Shenhe District, Shenyang, Liaoning 110016, China

## Abstract

It has been shown that inflammation and oxidative stress are important factors in postoperative atrial fibrillation (POAF). Angiotensin converting enzyme (ACE) and apelin have a close relationship with inflammation and oxidative stress. The effect of ACE and apelin on POAF after off-pump coronary artery bypass grafting (OPCABG) remains a question. The concentrations of serum ACE, angiotensin II (Ang II), apelin, bradykinin (BK), malondialdehyde (MDA), and C reactive protein (CRP) were measured in the perioperative period of OPCABG. The levels of serum ACE in the POAF group were higher than in the no POAF group both preoperatively and postoperatively. Apelin in the POAF group was lower than in the no POAF group. There was a correlation between serum ACE and apelin. Postoperatively, CRP and MDA in the POAF group were higher than in the no POAF group; however, there was no difference before the operation. Preoperative ACE and apelin were both significant and independent risk factors for POAF. In conclusion, the high ACE and low apelin preoperatively led to CRP and MDA being increased postoperatively, which was probably associated with POAF after OPCABG. Apelin may be a new predictor for POAF.

## 1. Introduction

**Figure 1:**
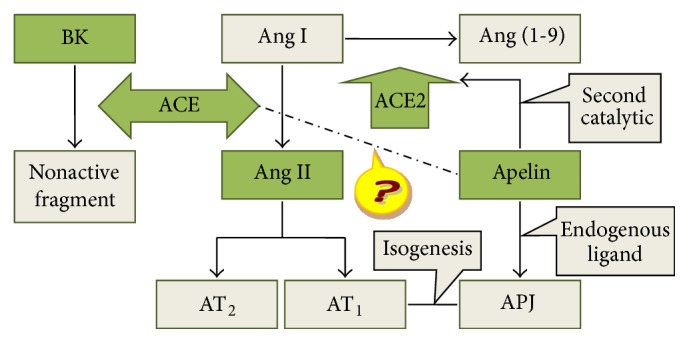
The relation between ACE, Ang II, BK, ACE2, and apelin.

**Figure 2:**
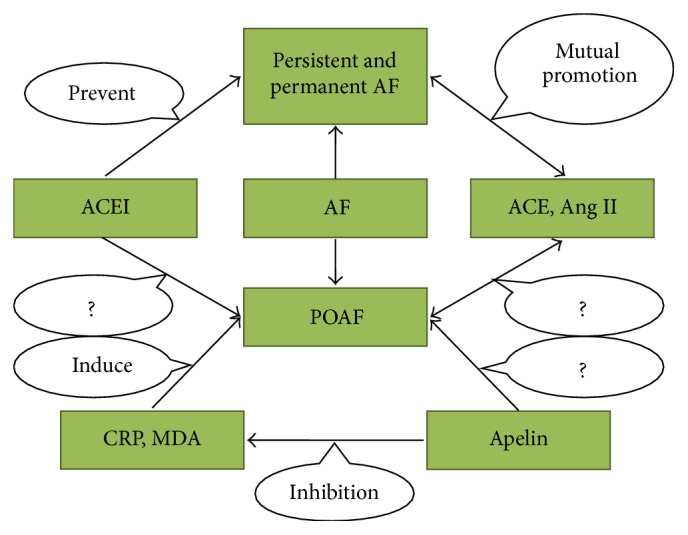
The relation between POAF, ACE, apelin, Ang II, ACEI, CRP, and MDA.

Postoperative atrial fibrillation (POAF) is a common postoperative complication following coronary artery bypass grafting (CABG), which occurs in approximately 13% to 40% of patients [1, 2]. POAF, as a type of new-onset paroxysmal atrial fibrillation (AF), is different from persistent and permanent AF in terms of mechanism. Existing research has shown that inflammation, oxidative stress, and electrical remodeling may trigger POAF without serious structural remodeling [3–5]. POAF induces hemodynamic instability in patients after the operation and is often accompanied by left-ventricular systolic dysfunction and congestive heart failure, increased risk of stroke, prolonged hospital stays, and increased medical costs and mortality rates [6]. It has been shown that the expressions of ACE and Ang II were increased in the atrial tissues of patients with persistent and permanent AF, which means ACEI can prevent the recurrence of persistent and permanent AF [7]. However, some studies found ACEI cannot prevent the occurrence of POAF [8], as it is known that ACE has a close relationship with Ang II and BK. Moreover, ACE has a negative correlation with angiotensin converting enzyme 2 (ACE2), which has a second catalytic substrate named apelin [9]. Apelin/APJ (a putative receptor protein related to the angiotensin receptor AT1) is a new member of RAAS discovered in recent years. Apelin has recently been demonstrated to regulate Ang II through ACE2. Apelin-ACE2-angiotensin 1-7 system has been proposed as a new network. A decrease in apelin causes downregulation of ACE2 expression, resulting in a reduction of Ang II degradation [10]. Several studies have shown that apelin has anti-inflammation and antioxidative stress effects [11–13]. However, the relationship between POAF, ACE, Ang II, BK, and apelin is not yet clear. We aimed to determine this relationship in the study and tried to find the new predictors for POAF (Figures 1 and 2). Maybe, the cause of ACEI not being able to prevent the occurrence of POAF would be found at the same time.

## 2. Methods/Design

### 2.1. Study Design

A retrospective case-controlled clinical trial was carried out. This clinical trial has been registered at ClinicalTrials.gov (registration number NCT02807532).

### 2.2. Study Setting

The study setting was Department of Cardiovascular Surgery, General Hospital of Shenyang Military Region, Shenyang, China.

### 2.3. Study Participants

A total of 508 patients with coronary atherosclerotic heart disease scheduled to undergo isolated OPCABG at our institution between March 2015 and December 2015 participated in the study. We only chose OPCABG to exclude the influence from on-pump CABG, similarly to blood dilution and trauma.

The inclusion criteria were the following: the subject met the WHO diagnostic criteria for coronary atherosclerotic heart disease, was scheduled to undergo OPCABG, had no history of atrial fibrillation, had no history of thoracotomy, and provided written informed consent having understood the benefits and risks of participation in the trial.

The exclusion criteria were the following: valvular heart disease requiring surgical treatment, on-pump CABG, malignant tumor, severe infection, chronic obstructive pulmonary disease (COPD), serious heart failure (EF < 0.30), refusal to cooperate with specimen collection and laboratory examination, ongoing participation in other clinical trials, and being unable to provide informed consent due to mental disorders or language barriers.

The concentrations of serum ACE, Ang II, apelin, BK, and MDA were measured using an enzyme linked immune sorbent assay (ELISA) and CRP was measured using immunoturbidimetry in the peripheral blood from 508 consecutive patients with a sinus rhythm in the perioperative period of OPCABG. Preoperative peripheral blood was obtained at 06:00-07:00 the morning after admission before the operation. Postoperative peripheral blood was obtained at 06:00-07:00 the morning after the operation. Both carried were out on an empty stomach.

### 2.4. POAF Definition and Monitoring

The patient's ECG in the intensive care unit (ICU) was continuously monitored for 48 hours after the operation. A standard 12-lead ECG was then recorded once per day after the patient returned to the general ward; ECGs were continuously monitored after any sign of clinical deterioration (palpitations, dyspnea, or confusion) until the POAF was converted. The above were measured for 7 days after the operation (or until the patient left the hospital). POAF was defined as a classical AF waveform lasting more than 1 minute.

### 2.5. Statistical Analysis

All statistical calculations were carried out using SPSS 18.0 software (IBM, Chicago, IL, USA). Continuous variables were reported as the mean ± standard deviation. Continuous variables with a normal distribution were analyzed with an independent *t*-test. Categorical variables were reported as frequency and percentage. Comparisons between groups were analyzed with a chi-squared test, correction chi-squared test, or Fisher's exact test. The continuous variables with an abnormal distribution were analyzed using the Mann–Whitney *U* test. Comparisons of data before and after the operation were evaluated using a paired *t*-test. The correlation between the 2 data sets was analyzed with bivariate correlation analysis. Independent risk factor analysis was used for univariate and multivariate logistic regression analysis. The factors where *P* < 0.1 were included in a multivariate logistic regression analysis as more factors were considered. *P* < 0.05 was considered significant in all comparisons.

## 3. Results

### 3.1. Cohort Characteristics

A total of 508 cases of OPCABG patients were included in this study. The incidence rate of POAF was 21.26% (108/508). The clinical characteristics of the patient groups in the POAF group and no POAF group are listed in Table 1. There were significant differences between the groups in age (*P* = 0.036), smoking (*P* = 0.044), left atrial diameters (LADs; *P* = 0.001), left ventricle end-diastolic volume (LVEDV; *P* = 0.013), and statins (*P* = 0.002).

### 3.2. Data Analysis of ACE, Apelin, Ang II, BK, CRP, and MDA

**Figure 3:**
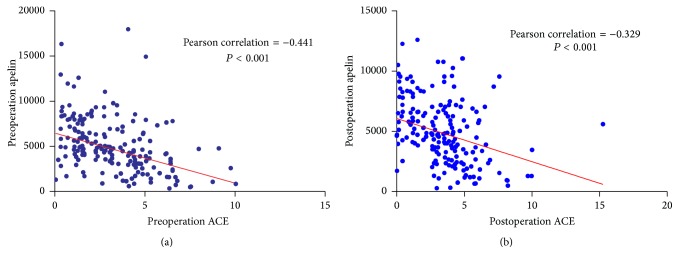
(a) Correlation analysis of preoperation ACE and apelin. There was correlation between serum ACE and apelin preoperatively (Pearson correlation = −0.441, *P* < 0.001). (b) Correlation analysis of postoperation ACE and apelin. There was correlation between serum ACE and apelin postoperatively (Pearson correlation = −0.329, *P* < 0.001).

**Figure 4:**
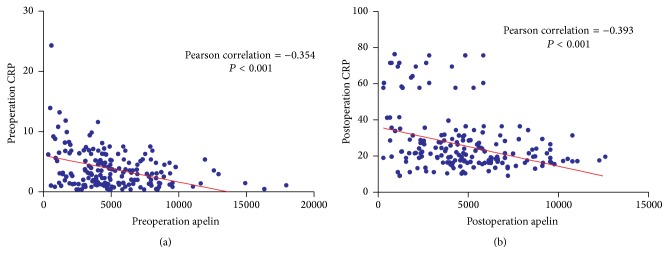
(a) Correlation analysis of preoperation apelin and CRP. There was a correlation between serum apelin and CRP preoperatively (Pearson correlation = −0.354, *P* < 0.001). (b) Correlation analysis of postoperation apelin and CRP. There was correlation between serum apelin and CRP postoperatively (Pearson correlation = −0.393, *P* < 0.001).

Data comparisons between the groups showed that the levels of serum ACE in the POAF group were higher than in the no POAF group both preoperatively and postoperatively (*P* = 0.001 and *P* = 0.033, resp.). The levels of serum apelin in the POAF group were lower than the no POAF group both preoperatively and postoperatively (*P* < 0.001, both). The levels of serum Ang II and BK showed no difference between the groups both preoperatively and postoperatively. The levels of CRP and serum MDA in the POAF group were higher than the no POAF group postoperatively (*P* = 0.005 and *P* = 0.002, resp.), but it showed no difference between the groups preoperatively. All the above results are listed in Table 2. There was a correlation between serum ACE and apelin preoperatively (Pearson correlation = −0.441, *P* < 0.001) and postoperatively (Pearson correlation = −0.329, *P* < 0.001; Figure 3). There was a correlation between serum apelin and CRP preoperatively (Pearson's correlation = −0.354, *P* < 0.001) and postoperatively (Pearson's correlation = −0.393, *P* < 0.001; Figure 4).

### 3.3. Univariate and Multivariate Analysis of POAF

Multivariate logistic regression analysis showed that ACE preoperatively (pre-ACE), pre-apelin, smoking, LDL, statins, EF, LAD, and LVEDV were significant and independent risk factors for POAF preoperatively (Wald = 8.197, OR = 1.188, 95% CI: 1.056–1.337, and *P* = 0.004; Wald = 7.366, OR = 0.485, 95% CI: 0.288–0.818, and *P* = 0.007; Wald = 8.755, OR = 2.172, 95% CI: 1.299–3.630, and *P* = 0.003; Wald = 4.092, OR = 1.342, 95% CI: 1.009–1.784, and *P* = 0.043; Wald = 12.671, OR = 0.424, 95% CI: 0.264–0.680, and *P* < 0.001; Wald = 4.883, OR = 0.002, 95% CI: 0.000–0.493, and *P* = 0.027; Wald = 18.350, OR = 1.167, 95% CI: 1.087–1.252, and *P* < 0.001; and Wald = 7.908, OR = 0.986, 95% CI: 0.976–0.996, and *P* = 0.005, resp.; Table 3).

Of special note, POAF had two classifications regarding counted data. The difference between the high and low values of apelin detection results was larger. Using continuous variables would affect the logistic regression analysis results and we therefore converted the results of apelin to counted data with a two-classification method. The samples were divided into high and low groups using the total sample mean value (4410 pg/mL) which was the boundary value. The logistic regression analysis was subsequently carried out.

### 3.4. Data Comparison between Preoperative and Postoperative Levels

**Figure 5:**
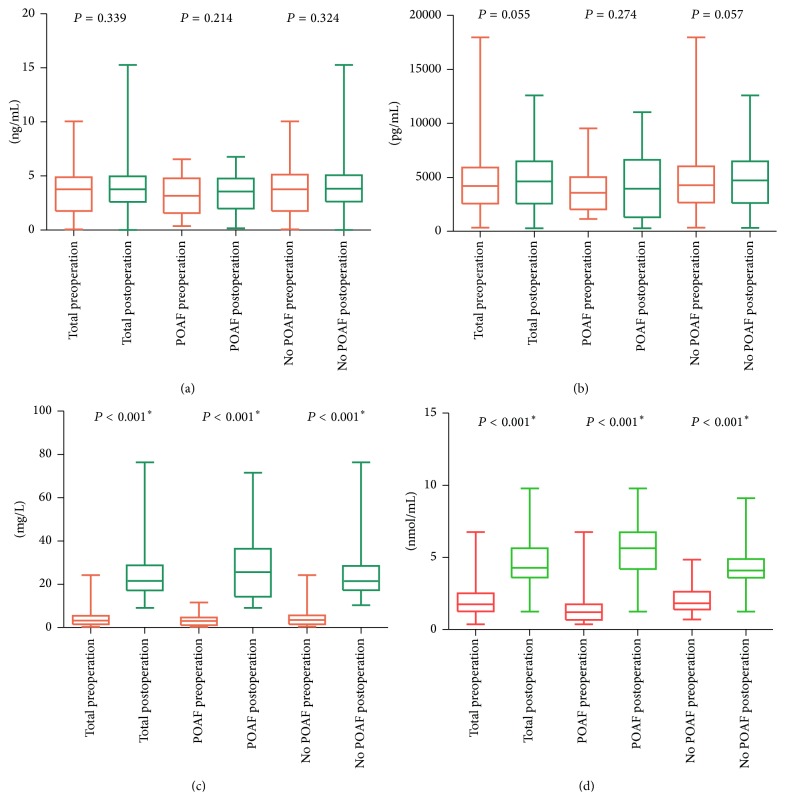
(a) A paired samples *t*-test was used to assess the levels of serum ACE preoperatively and postoperatively and they were not statistically significantly different in the entire sample (*P* = 0.339), the POAF group (*P* = 0.214), and the no POAF group (*P* = 0.324). (b) A paired samples *t*-test was used to determine the levels of serum apelin preoperatively and postoperatively and they were not statistically significantly different in the entire sample (*P* = 0.055), the POAF group (*P* = 0.274), and the no POAF group (*P* = 0.057). (c) A paired samples *t*-test was used to determine the levels of CRP between groups. The levels of CRP postoperatively were higher than preoperatively (^*∗*^*P* < 0.001, the results were statistically significant). (d) A paired samples *t*-test was used to determine the levels of MDA between groups. The levels of serum MDA postoperatively were higher than preoperatively (^*∗*^*P* < 0.001, the results were statistically significant).

Data comparisons between preoperative and postoperative levels showed that the levels of serum ACE, Ang II, apelin, and BK were not statistically significantly different in the entire sample, the POAF group, and the no POAF group. The levels of CRP and serum MDA postoperatively were higher than preoperatively (*P* < 0.001; Table 4 and Figure 5).

## 4. Discussion

The morbidity of POAF after CABG has been shown to be approximately 13% to 40% [1, 2]. It was 21.26% in our study. Several studies have shown that age, smoking, hypertension, diabetes (DM), obesity, hypercholesterolemia, leukocytosis, LAD, cardiopulmonary bypass time, and many other factors were found to be independent risk factors for POAF [14–16]. This study showed that ACE preoperatively (pre-ACE), pre-apelin, smoking, LDL, statins, EF, LAD, and LVEDV were significant and independent risk factors for POAF preoperatively. It is well known that myocardial fibrosis and structure reconstruction are important pathological bases of AF. Perhaps, there was a certain extent of myocardial fibrosis in patients with POAF before CABG. They were heavier than normal, but lighter than in persistent and permanent AF patients. When these patients had surgical trauma, postoperative inflammation and oxidative stress may trigger POAF. The sensitivity of the body to inflammation and oxidative stress is also one of the factors. EF, LAD, and LVEDV are closely related to myocardial fibrosis and structure reconstruction. Lower EF and LVEDV often suggested that there was obvious cardiac cavity enlargement and myocardial fibrosis. Larger LAD often suggested obvious structural reconstruction. These are exactly the bases of POAF. Smoking and statins are closely related to inflammation and oxidative stress. Anti-inflammatory and antioxidative effects of statins are already well known, as well as the negative effects of smoking [14–16]. These inferences are consistent with the results of this study.

The renin-angiotensin-aldosterone system (RAAS) is a hormone system that is involved in the regulation of the plasma sodium concentration and arterial blood pressure. It is an important control system in the human body. Several studies have shown that ACE and Ang II, as the major characters of RAAS, participate in the pathological process of POAF [7, 17–19]. Regarding the preventive effect of ACEI on POAF, a number of clinical studies have shown that the conclusions were not consistent [19–21].

Apelin is an endogenous ligand of the G-protein coupled receptor APJ, and it exhibits homology to angiotensin II. The signaling system stimulated by apelin regulates many physiological functions and pathological processes [22]. It is known that apelin has anti-inflammation and antioxidative stress effects [11–13, 23]. Existing research has shown that inflammation and oxidative stress are major pathogenic factors of POAF [3–5].

The relationships between POAF, ACE, Ang II, BK, and apelin are unknown. The aim of this study was to investigate this question. We measured the level of ACE, Ang II, apelin, BK, CRP, and MDA preoperatively and postoperatively in the POAF group and no POAF group. The conclusions were as follows.

First, the levels of serum ACE and apelin were significantly different between the POAF group and no POAF group both preoperatively and postoperatively, but the levels of serum Ang II and BK showed no difference. These data suggest that the predominant downstream effectors of ACE, Ang II, and BK may have little influence on the pathological processes of OPCABG-POAF. Perhaps this is the reason for ACEI not having a significant preventive effect on POAF. The OPCABG operation may not affect Ang II. Raised Ang II after persistent and permanent AF may be caused by myocardial fibrosis.

Second, there was a correlation between serum ACE and apelin preoperative and postoperative levels. At the same time, ACE and apelin were both significant and independent risk factors for POAF preoperatively. The results showed that ACE and apelin/APJ perhaps not only play important roles in OPCABG-POAF but also work together. It is known that ACE and ACE2 have a negative correlation in the human body. Apelin is a second catalytic substrate for ACE2 and functions as an inotropic and cardiovascular protective peptide [9]. The increased level of ACE perhaps reduces the level of apelin.

Third, we found that the influence of the operation on the level of ACE, Ang II, apelin, and BK was not obvious. The difference between preoperation and post-AF levels that has been shown in many studies may be due to POAF itself and may have nothing to do with OPCABG surgery. The higher level of ACE and the lower level of apelin preoperatively were the risk factors for POAF and were not changed after the operation. CRP and MDA are classical detection indices for inflammation and oxidative stress [5, 23, 24]. Increased levels of CRP and MDA after the operation showed the role of inflammation and oxidative stress perhaps more importantly. The reduced level of apelin and its anti-inflammation and antioxidative stress function may be one of the reasons for POAF. ACEI prevent the recurrence of persistent and permanent AF as it has antifibrosis effect but the main reason for the occurrence of POAF may be inflammation and oxidative stress.

In conclusion, we observed the changes and differences in levels of ACE, apelin, inflammation, and oxidative stress may be the biological reasons for POAF from this study. Higher serum ACE levels were perhaps a risk factor for POAF and higher serum apelin levels were perhaps a protection factor. Apelin may be a new predictor for POAF. The potential value of this information should be further evaluated in larger, prospective, multicenter studies.

## Figures and Tables

**Table 1:** Demographic and clinical characteristics by presence of POAF.

Characteristics	POAF group *n* = 108	No POAF group *n* = 400	*P* value
Sex (male/female)	80/28	300/100	0.844
Age (years)	63.51 ± 7.00	61.90 ± 7.10	0.036^*∗*^
BMI (kg/m^2^)	25.16 ± 2.73	24.80 ± 2.62	0.206
Smoking (%)	78 (72.2)	247 (61.8)	0.044^*∗*^
Heart rate (beats/min)	73.93 ± 8.50	72.37 ± 10.30	0.150
NYHA III-IV (%)	20 (18.5)	68 (17.0)	0.711
Hypertension (%)	76 (70.4)	252 (63.0)	0.155
DM (%)	48 (44.4)	172 (43.0)	0.788
Myocardial infarction (%)	62 (57.4)	203 (50.8)	0.161
Blood glucose (mmol/L)	6.38 ± 1.42	6.32 ± 1.77	0.758
Cholesterol (mmol/L)	4.38 ± 1.06	4.31 ± 0.92	0.514
Triglyceride (mmol/L)	2.03 ± 0.63	2.03 ± 1.30	0.982
LDL (mmol/L)	2.75 ± 0.87	2.58 ± 0.83	0.053
HDL (mmol/L)	0.93 ± 0.10	0.95 ± 0.16	0.144
Creatinine (umol/L)	73.14 ± 12.20	72.59 ± 14.92	0.724
CKMB (U/L)	10.10 ± 7.11	9.71 ± 6.69	0.592
cTNT (ng/mL)	0.10 ± 0.14	0.07 ± 0.12	0.101
EF	0.54 ± 0.05	0.55 ± 0.05	0.064
LAD (mm)	38.41 ± 5.48	36.62 ± 4.52	0.001^*∗*^
PASP (mmHg)	35.26 ± 13.35	33.99 ± 6.06	0.152
LVEDV (mL)	120.89 ± 48.16	110.36 ± 36.34	0.013^*∗*^
Beta-blockers (%)	97 (89.8)	368 (92.0)	0.469
ACEI (%)	44 (40.7)	184 (46.0)	0.330
CCB (%)	60 (55.6)	204 (51.0)	0.400
Statins (%)	60 (55.6)	284 (71.0)	0.002^*∗*^

*Notes*. Data presented as mean ± standard deviation or as a ratio. ^*∗*^*P* value < 0.05; the difference was statistically significant. POAF, postoperative atrial fibrillation; BMI, body mass index; NYHA, New York Heart Association; DM, diabetes mellitus; LDL, low-density lipoprotein; HDL, high-density lipoprotein; CKMB, creatine kinase MB; EF, ejection fraction; LAD, left atrial diameter; PASP, pulmonary arterial systolic pressure; LVEDV, left ventricle end-diastolic volume; ACEI, angiotensin converting enzyme inhibitor; CCB, calcium channel blocker.

**Table 2:** Comparison of the differences of serum ACE, Ang II, BK, apelin, CRP, and MDA between the POAF group and no POAF group.

Characteristics	POAF group *n* = 108	No POAF group *n* = 400	*P* value
Pre-ACE (ng/mL)	4.31 ± 1.94	3.53 ± 2.16	0.001^*∗*^
Post-ACE (ng/mL)	4.34 ± 2.12	3.77 ± 2.50	0.033^*∗*^
Pre-Ang II (pg/mL)	242.07 ± 149.97	254.87 ± 131.82	0.385
Post-Ang II (pg/mL)	246.33 ± 138.36	239.34 ± 135.75	0.636
Pre-BK (pg/mL)	1772.98 ± 673.56	1754.19 ± 946.09	0.847
Post-BK (pg/mL)	1685.53 ± 792.97	1739.74 ± 909.94	0.573
Pre-apelin (pg/mL)	3171.92 ± 2207.75	4745.24 ± 2718.03	<0.001^*∗*^
Post-apelin (pg/mL)	3872.33 ± 2954.44	4881.90 ± 2487.06	<0.001^*∗*^
Pre-CRP (mg/L)	4.52 ± 2.88	3.99 ± 3.48	0.146
Post-CRP (mg/L)	29.35 ± 19.10	24.98 ± 12.68	0.005^*∗*^
Pre-MDA (nmol/mL)	2.16 ± 1.28	1.98 ± 1.09	0.140
Post-MDA (nmol/mL)	5.09 ± 2.11	4.48 ± 1.68	0.002^*∗*^

*Notes*. Data presented as mean ± standard deviation. ^*∗*^*P* value < 0.05; the difference was statistically significant. ACE, angiotensin converting enzyme; Ang II, angiotensin II; BK, bradykinin; CRP, C reactive protein; MDA, malondialdehyde; Pre, preoperation; Post, postoperation. Other abbreviations are as in Table 1.

**Table 3:** Multivariate logistic regression analysis of preoperation risk factors of POAF in 508 cases overall sample.

Variable	Wald-value	OR (95% CI)	*P* value
Age	2.449	1.028 (0.993–1.065)	0.118
Smoking	8.755	2.172 (1.299–3.630)	0.003^*∗*^
LDL	4.092	1.342 (1.009–1.784)	0.043^*∗*^
Statins	12.671	0.424 (0.264–0.680)	<0.001^*∗*^
EF	4.883	0.002 (0.000–0.493)	0.027^*∗*^
LAD	18.350	1.167 (1.087–1.252)	<0.001^*∗*^
LVEDV	7.908	0.986 (0.976–0.996)	0.005^*∗*^
Pre-ACE	8.197	1.188 (1.056–1.337)	0.004^*∗*^
Pre-apelin^*∗*^	7.366	0.485 (0.288–0.818)	0.007^*∗*^

*Notes*. OR, odds ratio; CI, confidence interval; ^*∗*^*P* value < 0.05. Other abbreviations are as in Tables 1 and 2.

**Table 4:** Comparison of the differences of serum ACE, Ang II, BK, apelin, CRP, and MDA between preoperation and postoperation in each group.

Characteristics	Preoperation	Postoperation	*t*-value	*P* value
*Total n* = 508				
ACE (ng/mL)	3.70 ± 2.14	3.81 ± 2.39	−0.957	0.339
Ang II (pg/mL)	252.15 ± 135.83	254.71 ± 140.90	−0.312	0.755
BK (pg/mL)	1758.19 ± 894.56	1728.21 ± 885.90	0.700	0.484
Apelin (pg/mL)	4410.76 ± 2694.02	4696.95 ± 2598.99	−1.922	0.055
Crp (mg/L)	4.10 ± 3.36	25.91 ± 14.38	−40.215	<0.001^*∗*^
MDA (nmol/mL)	2.02 ± 1.14	4.61 ± 1.79	−32.463	<0.001^*∗*^
*POAF group n* = 108				
ACE (ng/mL)	3.29 ± 1.62	3.43 ± 1.73	−1.251	0.214
Ang II (pg/mL)	284.73 ± 151.76	278.56 ± 156.00	0.487	0.627
BK (pg/mL)	1772.98 ± 673.56	1685.53 ± 792.97	0.850	0.397
Apelin (pg/mL)	4029.90 ± 2277.83	4296.88 ± 2918.46	−1.100	0.274
Crp (mg/L)	3.37 ± 2.40	29.50 ± 18.35	−15.733	<0.001^*∗*^
MDA (nmol/mL)	1.47 ± 1.61	5.48 ± 1.95	−22.208	<0.001^*∗*^
*No POAF group n* = 400				
ACE (ng/mL)	3.81 ± 2.25	3.91 ± 2.53	−0.986	0.324
Ang II (pg/mL)	243.32 ± 130.03	248.28 ± 136.03	−0.574	0.567
BK (pg/mL)	1754.19 ± 946.09	1739.74 ± 909.94	0.309	0.758
Apelin (pg/mL)	4520.87 ± 2784.43	4804.97 ± 2498.79	−1.912	0.057
Crp (mg/L)	4.29 ± 3.55	24.85 ± 12.76	−41.351	<0.001^*∗*^
MDA (nmol/mL)	2.15 ± 1.04	4.37 ± 1.66	−28.177	<0.001^*∗*^

*Notes*. Data presented as mean ± standard deviation. ^*∗*^*P* value < 0.05; the difference was statistically significant. Other abbreviations are as in Tables 1 and 2.

## Additional Points

*Limitation*. This study included a retrospective and observational analysis of single-center data. The relatively small sample size in this study limited our capacity to statistically detect interactions between RAAS, apelin/APJ, and POAF. Further prospective studies would allow confirmation of these initial findings with the use of a larger sample size. Serum biomarker levels are really different from local (atrial) levels. However, collection of myocardial tissue is not routine in OPCABG. It can affect the integrity of the patient's heart and most patients reject this. This is different from valve replacement surgery, which is another limitation in this research. The rhythm follow-up of patients after 48 h was limited based on clinical considerations but could result in an underestimate of POAF cases. Furthermore, the striking OR of some clinical variables (e.g., EF) requires further investigation in future studies.
